# Diagnostic Efficacy of RealStar SARS-CoV-2 Reverse Transcription-Polymerase Chain Reaction (RT-PCR) in Comparison to GeneXpert System for the Detection of COVID-19

**DOI:** 10.7759/cureus.35158

**Published:** 2023-02-18

**Authors:** Dunia M Jawdat, Gadah S Aljarallah, Maha A Albakr, Reema M Alajlan, Reem F Almesfir, Nada F Alqubaibi, Maram M Albalawi, Ali A Alshehri, Sameera M Aljohani

**Affiliations:** 1 Cellular Therapy Services, King Abdullah International Medical Research Center, Riyadh, SAU; 2 College of Medicine, King Saud Bin Abdulaziz University for Health Sciences, Riyadh, SAU; 3 Biostatistics, King Abdullah International Medical Research Center, Riyadh, SAU; 4 Pathology and Laboratory Medicine, King Abdulaziz Medical City, Riyadh, SAU; 5 Infectious Diseases Research Department, King Abdullah International Medical Research Center, Riyadh, SAU

**Keywords:** saudi arabia, realstar® sars-cov-2 rt–pcr, genexpert®, covid-19, acute respiratory illness (ari) score

## Abstract

Background and objective

The coronavirus disease 2019 (COVID-19) pandemic has become a major health concern due to the rapid transmission of the virus that causes it: severe acute respiratory syndrome coronavirus 2 (SARS-CoV-2). To address the growing demand on healthcare systems to control this pandemic, more effective diagnostic methods need to be applied. In this study, we aimed to compare the efficacy of RealStar® SARS-CoV-2 reverse transcription-polymerase chain reaction (RT-PCR) versus the GeneXpert® system.

Methods

A retrospective cross-sectional study was conducted in the central lab of King Abdulaziz Medical City (KAMC) in Riyadh, Saudi Arabia. Data from all nasopharyngeal swabs (NPS) (150,000) submitted for SARS-CoV-2 analysis from July 2020 to July 2021 were reviewed retrospectively. Furthermore, all NPS (n=384) that were analyzed on both the RealStar® SARS-CoV-2 RT-PCR and GeneXpert® systems for confirmatory purposes were included in the study. Acute respiratory illness (ARI) screening forms of the selected samples were reviewed from the electronic database (BestCare system), and they were analyzed and compared at one point in time; therefore, a cross-sectional study was found to be the best suitable study design. Using the statistical analysis software, the receiver operating characteristic (ROC) curve was obtained to compare the sensitivity (Sn), specificity (Sp), positive predictive value (PPV), and negative predictive value (NPV). The test was considered significant if the area under the curve (AUC) value was >0.5.

Results

The diagnostic performance of the RealStar® and GeneXpert® assays in detecting SARS-CoV-2 was evaluated using ROC curve analysis, which showed AUCs of 0.597 and 0.637, respectively. In addition, 35% of the total results fell into a substantial agreement of 0.76 (95% CI: 0.6626-0.8732). The majority of the NPS were reported negative by both RealStar® (246, 80.66%) and GeneXpert® (226, 74.10%). Most samples (210, 68.85%) were obtained from asymptomatic patients, scoring less than 4 (ARI <4) based on the ARI screening form.

Conclusion

Based on the AUC of ROC, there is no significant difference in the performance characteristics between the RealStar® RT-PCR and GeneXpert® in detecting COVID-19.

## Introduction

Coronaviridae is a family of the most common large, enveloped, and single-stranded RNA respiratory viruses [1]. It comprises a wide variety of viruses that can cause symptoms ranging from the common cold to more serious ailments [2,3]. A few types of coronaviruses have contributed to multiple public health outbreaks around the world. For example, the severe acute respiratory syndrome coronavirus 2 (SARS-CoV-2), which was first reported in late December 2019, has caused highly contagious viral illnesses leading to a pandemic known as coronavirus disease 2019 (COVID-19) [4,5].

The World Health Organization (WHO) has stated that hundreds of thousands of COVID-19 cases have been confirmed in Saudi Arabia, illustrating its local significance [6]. This contagious viral illness has not only had tragic effects on Saudi Arabia but also on the whole world, causing millions of fatalities worldwide [7]. Government authorities in most countries around the globe have taken major steps and preventative measures to contain and prevent SARS-CoV-2 transmission [8]. The Kingdom of Saudi Arabia has been one of the countries to implement several preventative measures to decelerate the spread of the virus. In addition, both the Saudi government and its private sector have collaborated to create and launch multiple applications and platforms to carry out public health duties and provide healthcare services [9,10]. However, all these strict precautions due to COVID-19 cases have negatively impacted the country's economy, consequently affecting the healthcare sector and causing shortages in medical supplies, including COVID-19 PCR kits [11,12]. This impact has resulted in a more challenging environment for healthcare workers and calls for a more efficient diagnostic method to control this pandemic more easily.

The different coronavirus-related outbreaks have led to the development of many diagnostic tests to determine the spread of the virus [5]. The common methods of testing viruses and SARS-CoV-2 specifically are categorized as serology tests and nucleic acid amplification tests (NAATs) [13]. NAAT involves isothermal amplification technologies and reverse transcription-polymerase chain reaction (RT-PCR) [13]. Quantitative RT-PCR is the most widely used NAAT test [14]. The major genes and the primary choices for forming antigens in SARS-CoV-2 are the N and S structural proteins; therefore, they are the main targets for SARS-CoV-2 testing [14]. It is currently recommended to combine different SARS-CoV-2 RT-PCR panel assays, with multiple targets, to reduce the possibility of sensitivity loss caused by protein S mutations [15].

As a response to the initial outbreak, detection tests were important to enhance containment and mitigation strategies and avoid uncontrolled viral spread [5]. Therefore, several kits have been developed specifically to detect the novel COVID-19. The central lab of King Abdulaziz Medical City (KAMC) in Riyadh utilizes two main kits: the GeneXpert® kit and the RealStar® SARS-CoV-2 RT-PCR kit. The S and E genes are targeted by the RealStar® SARS-CoV-2 RT-PCR kit as specific genes for COVID-19 [16]. According to Visseaux et al. [17], the RealStar® assay demonstrated a sensitivity of 97.8% and a specificity of 97.3%. The test can detect small levels of virus regardless of the presence of symptoms; in contrast, sample degradation, low quality of specimen collection, time-consuming nature, and the low efficacy of some PCR kits can increase false negatives [18]. GeneXpert® is another kit that works through RNA amplification; a systemic review with a meta-analysis has proven its efficacy by demonstrating a sensitivity of 100% and specificity of 80% [19]. Conversely, Al-kindi et al. [20] have stated that GeneXpert® has detected diverse SARS-CoV-2-specific genes (E and N), which may lead to false-negative results if a mutation prevents primer binding.

The primary stages of providing patient identification, segregation, and optimal interventions depend on the accessibility and validity of reliable bioassays that provide sensitive recognition of infectious agents [21]. With the increased demand on healthcare systems to prevent and control this pandemic, and based on Shyu et al. [22], more effective diagnostic methods for COVID-19 should be applied.

Due to the lack of comparative analyses related to this topic in the literature, we conducted a retrospective cross-sectional study to assess the performance and diagnostic efficacy of RealStar® SARS-CoV-2 RT-PCR compared to those of the GeneXpert® system as a diagnostic method for COVID-19 based on nasopharyngeal swabs (NPS) sent to King AbdulAziz Medical Lab, Riyadh, Saudi Arabia. This comparative study will attempt to assess the efficacies of the two tests, aiming to provide more insights into the diagnostic process.

This article was previously presented as a poster abstract at the 2022 Health Professions Conference in Riyadh, Saudi Arabia, on December 26, 2022.

## Materials and methods

Study setting and participants

This retrospective cross-sectional study, conducted in the central lab of KAMC in Riyadh, Saudi Arabia, aimed to compare the diagnostic accuracy between RealStar® SARS-CoV-2 RT-PCR and GeneXpert® in detecting SARS-CoV-2. Data from all NPS (150,000) submitted for SARS-CoV-2 analysis from July 2020 to July 2021 were reviewed retrospectively. Furthermore, all NPS data (n=384) that were analyzed in both the RealStar® SARS-CoV-2 RT-PCR and GeneXpert® systems for confirmatory purposes were included in the study. Acute respiratory illness (ARI) screening forms of the selected samples were reviewed from the electronic database (BestCare system), and they were analyzed and compared at one point in time; therefore, a cross-sectional study was found to be the best suitable study design.

Data collection method

Chart review was the method employed in this study, and the independent variables assessed were the ARI score, GeneXpert® test results, and RealStar® SARS-CoV-2 RT-PCR test results. The ARI score was computed based on the risk of exposure and the signs and symptoms that were obtained from the patient [19]. The ARI score data along with both kits' results were obtained from the BestCare laboratory system, and it was documented in a Microsoft Excel sheet with a serial number. The ARI screening forms for the selected samples were coded as 1 if the ARI was ≥4, indicating symptomatic patients, and 0 if the ARI was <4, indicating asymptomatic patients (Appendix 1). In addition, the kit results were coded in the data collection sheet as 1 for positive results and 0 for negative results. Both test results and the ARI score of the same patient were used to determine the false positive (FP), false negative (FN), true positive (TP), and true negative (TN) (if the sample's result was positive and the ARI was ≥4: TP; if the sample's result was negative and the ARI was <4: TN).

Using this data, a two-by-two table was generated to obtain the dependent variables: the sensitivity (Sn), specificity (Sp), positive predictive value (PPV), and negative predictive value (NPV) of each kit. Patient credibility when answering the queries related to ARI score, as well as sample collection, and processing were the confounder variables that could affect the viral overload and, consequently, affect the dependent variables. To avoid any verification or referral bias, the 384 swabs tested for both RealStar® SARS-CoV-2 RT-PCR and GeneXpert, yielding a total of 768 results, were from two separate teams that were blinded to each other's test results.

Data analysis

After collecting each test result, each sample was labeled with a serial number using Excel 2016. The variables for each test were transferred properly as numerical data to the variable sheets of the Statistical Analytics Software (SAS, version 9.4) to be further used for analysis. The receiver operating characteristic (ROC) curve was used to compare the Sn, Sp, PPV, and NPV for each test, and it is presented as a percentage. Additionally, the area under the curve (AUC) has been reported, and the test is considered significant if the AUC value is >0.5. Moreover, SAS (version 9.4) was used to represent the degree of agreement between RealStar® SARS-CoV-2 RT-PCR and the GeneXpert® system based on the mean difference and standard deviation (SD) of the positive results. In addition, all patient information related to their samples was confidential, and only the lab workers and research team had access to it. The anonymity of the participants was maintained throughout the study.

## Results

This study was conducted on 384 NPS collected from KAMC central lab during the period from July 2020 to July 2021. Each sample was tested by RT-PCR using both the RealStar® and GeneXpert® kits, yielding 768 results. Of the 768 test results, 158 (79 samples) were excluded from the analysis either due to a missing test result or a missing ARI score. Most of the NPS were reported negative by both RealStar® and GeneXpert®. Of the tested samples, 16 and 26 were found inconclusive by the RealStar® and GeneXpert®, respectively (Table 1). These inconclusive data were also excluded. Comparing the two systems, RealStar® showed a higher TN score than GeneXpert® but a lower TP score of 26 (28.89%) (Table 2). Most of the samples (210, 68.85%) were obtained from asymptomatic patients, scoring less than 4 (ARI <4) based on the ARI screening form (Table 3). Furthermore, 279 (GeneXpert® kit) and 289 (RealStar® kit) samples were used for further descriptive and inferential statistical analysis.

**Table 1:** RealStar® assay versus GeneXpert® assay diagnostic performance for detecting SARS-CoV-2 SARS-CoV-2: severe acute respiratory syndrome coronavirus 2

	RealStar® (n=305), n (%)	GeneXpert® (n=305), n (%)
Total positives	43 (14.10%)	53 (17.38%)
Total negatives	246 (80.66%)	226 (74.10%)
Total inconclusive	16 (5.25%)	26 (8.52%)
Total conclusive	289 (94.75%)	279 (91.47%)

**Table 2:** RealStar® assay versus GeneXpert® assay diagnostic performance for detecting SARS-CoV-2 SARS-CoV-2: severe acute respiratory syndrome coronavirus 2

	RealStar® (n=289)	GeneXpert® (n=279)
True positive	26 (28.89%)	28 (30.43%)
False positive	17 (8.54%)	25 (13.37%)
True negative	182 (91.46%)	162 (86.63%)
False negative	64 (71.11%)	64 (69.57%)
Sensitivity	28.89%	30.43%
Specificity	91.46%	86.63%
Positive predictive value	60.47%	52.83%
Negative predictive value	73.98%	71.68%

**Table 3:** Acute respiratory illness (ARI) screening score results

ARI screening	Frequency	Percent
Asymptomatic	210	68.85%
Symptomatic	95	31.15%

Descriptive statistics

The analysis reported many similarities between the diagnostic performance outcomes of both the RealStar® and GeneXpert® assays. The RealStar® kit showed a sensitivity of 28.8% (95% CI: 0.1952-0.3825) and a specificity of 91% (95% CI: 0.8757-0.9534). In contrast, the GeneXpert® kit showed a sensitivity of 30.4% (95% CI: 0.2103-0.3984) and a specificity of 86.6% (95% CI: 0.8175-0.9151). However, GeneXpert® showed a lower NPV and PPV than RealStar® (Table 2).

Inferential statistics

**Figure 1:**
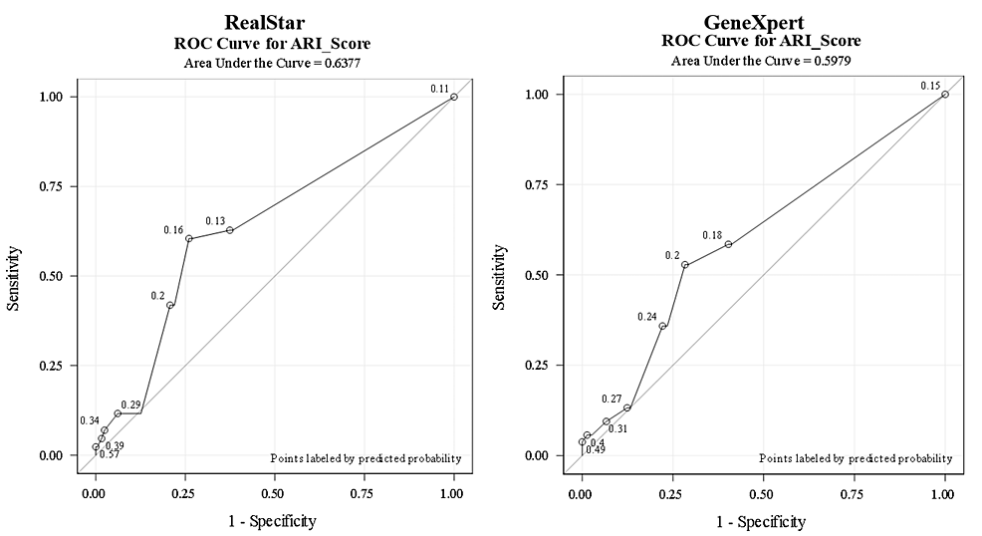
ROC curve analysis for the RealStar® assay and GeneXpert® assay ROC: receiver operating characteristic; ARI: acute respiratory illness

**Figure 2:**
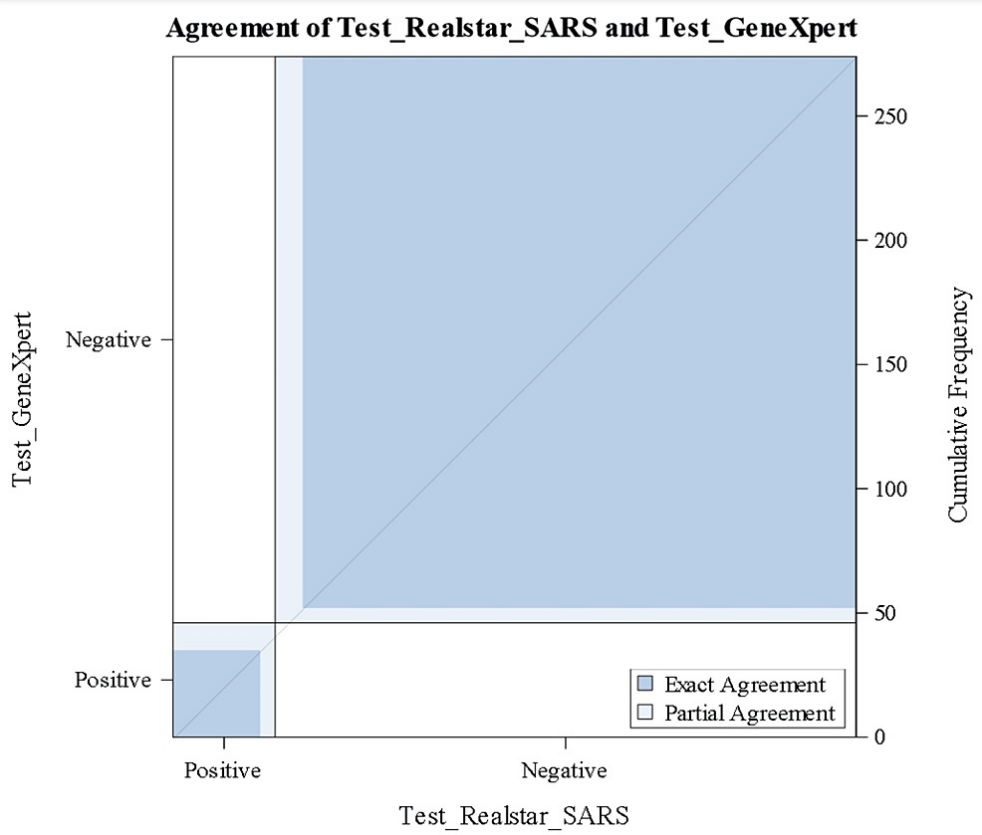
Cohen's kappa level of agreement for RealStar® and GeneXpert®

The ROC curve analysis was used for the diagnostic performance of both RealStar® and GeneXpert® assays in detecting SARS-CoV-2 and revealed AUCs of 0.597 and 0.637, respectively. There was no significant difference and no class separation between both methods (Figure 1). Of all test results, 35% fell into a substantial agreement of 0.76 (95% CI: 0.6626-0.8732) (Figure 2).

## Discussion

The purpose of this study was to compare the performances of RealStar® and GeneXpert® in detecting COVID-19. The findings showed many similarities between RealStar® and GeneXpert® in detecting COVID-19. Both had similar sensitivity; however, RealStar® had slightly higher specificity than GeneXpert® (Table 2).

Rakotosamimanana et al. [19] found the GeneXpert® specificity to be 80%, which is in approximate agreement with our findings. On the contrary, GeneXpert® pooled a sensitivity of 100%, which was found to be much lower in our results [19]. This disparity could be attributed to self-reported ARIs or to patients infected with other viruses with clinical presentations and symptoms similar to COVID-19. Moreover, Singh and Dhibar [23] found that the specificity and sensitivity of GeneXpert® in detecting COVID-19 were 99% and 88%, respectively. These were also higher than our study’s findings. Such differences could be related to variances in the tested specimens. In our study, we included only nasopharyngeal specimens, in contrast to the study by Singh and Dhibar [23], where they included a variety of specimens (Table 2).

Furthermore, Visseaux et al. [17] demonstrated the sensitivity and specificity of the RealStar®️ SARS-CoV-2 RT-PCR as 97.8% and 97.3%, respectively, whereas our study showed lower results. In addition, Schneider et al. [24] studied 103 samples that were pre-tested by RealStar®️ SARS-CoV-2 RT-PCR, and they found a positive percentage agreement (sensitivity) of 95.7% and a negative percentage agreement (specificity) of 100%. Both studies generated much higher results than ours, which might be due to the detection of different genome areas; they targeted the viral E gene and S gene, whereas, in our study, the KAMC central lab only focuses on extracting the S gene [17,24]. Moreover, the sensitivity and specificity of test results often vary according to the disease prevalence in the conducted area [25] (Table 2).

Since this study employed a cross-sectional design, it led to the analysis of fewer samples than anticipated. Furthermore, the insufficient number of samples that were tested in both kits was due to our specified time frame and the exclusion of both inconclusive samples and samples with missing ARIs. These two points highlight the limitations of our study. Because the cross-sectional study design has this limitation, we suggest that it would be preferable to use a longitudinal prospective study design to be able to test each sample with both kits using their respective complete ARI documents.

In summary, this study showed comparable performance between the two kits that we used for testing. Additionally, the advantage of RealStar® over GeneXpert® lies in its ability to process a larger number of samples simultaneously, making it the best option for mass-scale testing, whereas GeneXpert® can be lifesaving in emergencies [24]. Therefore, because GeneXpert® has a higher sensitivity, it could be used to rule out COVID-19, meaning it could be employed as the first-line method in detecting COVID-19, whereas RealStar® could be used to confirm COVID-19.

## Conclusions

According to the AUC of ROC, the findings of this study indicate that there is no significant difference between the performance characteristics of RealStar® and GeneXpert® in detecting COVID-19. Moreover, these two kits could be used interchangeably. We recommend that future studies compare the two kits in a more comprehensive and detailed manner, including their cost-effectiveness and viral load documentation, as well as analyzing the association of other types of respiratory specimens, such as sputum and bronchial lavage, in manipulating the results.

## Figures and Tables

**Figure 3:**
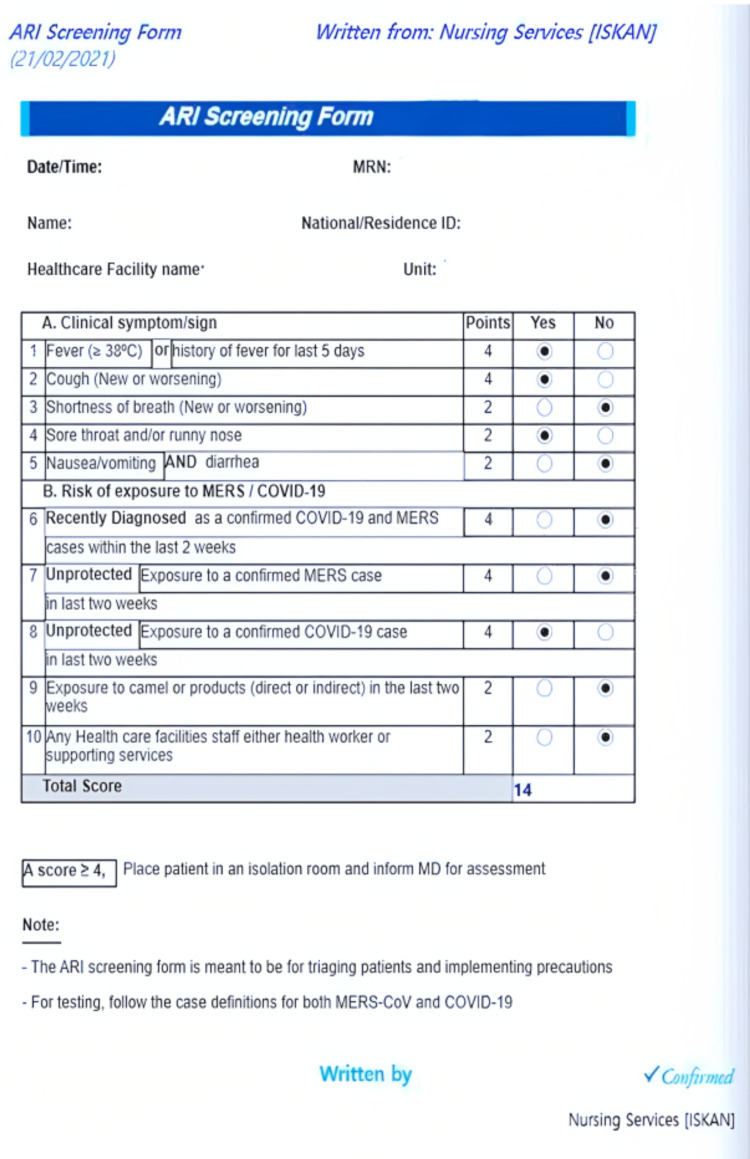
Acute respiratory illness (ARI) screening form
